# Elevated Pre-Treatment Serum MMP-7 Levels Are Associated with the Presence of Metastasis and Poor Survival in Upper Tract Urothelial Carcinoma

**DOI:** 10.3390/biomedicines10030698

**Published:** 2022-03-17

**Authors:** Petra Terézia Kovács, Tamás Mayer, Anita Csizmarik, Melinda Váradi, Csilla Oláh, Ádám Széles, Stephan Tschirdewahn, Ulrich Krafft, Boris Hadaschik, Péter Nyirády, Péter Riesz, Tibor Szarvas

**Affiliations:** 1 Department of Urology, Semmelweis University, 1082 Budapest, Hungary; kovacs.petra@med.semmelweis-univ.hu (P.T.K.); mayer.tam96@gmail.com (T.M.); csizmarik.anita@gmail.com (A.C.); varadi9412@gmail.com (M.V.); szelesadam95@gmail.com (Á.S.); nyirady.peter@med.semmelweis-univ.hu (P.N.); rieszp@gmail.com (P.R.); 2 Department of Urology, University of Duisburg-Essen and German Cancer Consortium (DKTK)-University Hospital Essen, D-45147 Essen, Germany; olahcsilla5@gmail.com (C.O.); stephan.tschirdewahn@uk-essen.de (S.T.); ulrich.krafft@uk-essen.de (U.K.); boris.hadaschik@uk-essen.de (B.H.)

**Keywords:** upper urinary tract cancer, UTUC, MMP-7, matrix metalloproteinase, biomarker, prognosis, immune checkpoint inhibitor therapy, chemotherapy, radical nephroureterectomy

## Abstract

Upper tract urothelial carcinoma (UTUC) is a rare cancer with a barely predictable clinical behaviour. Serum MMP-7 is a validated prognostic marker in urothelial bladder cancer, a tumour entity with large clinical, histological, and molecular similarity to UTUC. The serum MMP-7 levels have not yet been investigated in UTUC. In the present study, we determined MMP-7 concentrations in an overall number of 103 serum samples from 57 UTUC patients who underwent surgical or systemic (platinum or immune checkpoint inhibitor) therapy by using the ELISA method. In addition to pre-treatment samples, the serum samples collected at predefined time points after or during therapy were also investigated. Serum MMP-7 concentrations were correlated with clinicopathological and follow-up data. Our results revealed significantly, two-fold elevated pre-treatment serum MMP-7 levels in metastatic cases of UTUC in both the radical surgery- and the chemotherapy-treated cohorts (*p* = 0.045 and *p* = 0.040, respectively). In addition, high serum MMP-7 levels significantly decreased after radical surgery, and high pre-treatment MMP-7 concentrations were associated with shorter survival both in the surgery- and chemotherapy-treated cohorts (*p* = 0.029 and *p* = 0.001, respectively). Our results revealed pre-treatment serum MMP-7 as a prognostic marker for UTUC, which may help to improve preoperative risk-stratification and thereby improve therapeutic decision-making.

## 1. Introduction

Urothelial carcinoma can develop in the entire urinary tract, including the proximal part of the urethra, bladder, ureters, and the renal pelvis. While approximately 90% of urothelial carcinomas are found in the urinary bladder, only 5–10% of cases are located in the upper urinary tract (ureters or renal pelvis) [1]. According to the data of Globocan, 573,278 new cases of bladder cancer (BC) were diagnosed worldwide in 2020, while no data were reported on the incidence of upper tract urothelial carcinoma (UTUC) [2]. In Western countries, the incidence of UTUC is 2/100,000 cases a year [1]. UTUC has long been considered as a rare localization of a common cancer, but in the last few years, a growing body of evidence has suggested clinical and molecular differences between BC and UTUC [3]. Accordingly, the European Association of Urology has published separate guidelines for BC and UTUC since 2011 [3]. The gold standard treatment for UTUC is radical nephroureterectomy (RNU) with or without perioperative (neoadjuvant or adjuvant) platinum-based chemotherapy and checkpoint inhibitor treatment for chemotherapy resistant or ineligible patients [4]. In selected, low-risk cases organ-sparing surgery is possible. Because of the difficult anatomical features of the upper urinary tract, the accuracy of the diagnostic transurethral biopsy of UTUC is limited, which may lead to overtreatment for some UTUC patients and undertreatment for others, and therefore represents therefore a significant clinical problem [5]. Preoperative prediction of pathological stage or patients’ prognosis may select patients for nephron-sparing surgery and therefore may prevent overtreatment. On the other hand, a more accurate prognostic stratification may identify high-risk patients who will benefit from a more aggressive perioperative systemic treatment. Therefore, preoperative risk-stratification of UTUC may significantly improve the quality of therapeutic decision-making and the prognosis of patients. Currently, no biomarkers are available for the preoperative risk-stratification of UTUC.

Matrix metalloproteinases (MMPs) are a family of calcium-dependent, zinc-containing proteolytic enzymes, which are capable of proteolytically cleaving all components of the extracellular matrix, such as collagens, laminin, fibronectin, and gelatine. In addition, MMPs are involved in several tumour related processes, including apoptosis, angiogenesis, and epithelial-to-mesenchymal transition. MMPs are involved in both physiological (reproduction, tissue remodelling, etc.) and pathological processes (tumour progression, metastasis, etc.) [6,7]. MMP-7, also known as matrilysin, is the smallest member of the MMP family (18 kDa size), which is considered to be directly secreted by tumour cells, in contrast to other members of the family, which are secreted by stromal cells (e.g., fibroblasts or macrophages). We previously found preoperative serum and urine MMP-7 levels to be significantly associated with a higher tumour stage and the presence of lymph node metastases in BC [7,8]. Accordingly, BC patients with elevated preoperative serum MMP-7 levels had a significantly shorter survival [7]. This observation could be confirmed in several independent BC patient cohorts [9,10,11,12]. The prognostic value of serum MMP-7 has not yet been analysed in UTUC. Therefore, in this study, we assessed pre-treatment and post-/on-treatment serum MMP-7 levels of UTUC patients who underwent surgical or systemic (platinum-based or immune checkpoint inhibitor) treatment, and correlated the results with the patients’ clinicopathological and follow-up data.

## 2. Materials and Methods

### 2.1. Patient Cohort

This study included a total of 103 serum samples from 57 UTUC patients (40 males and 17 females) who underwent surgical (RNU cohort; n = 34), systemic platinum (CTX cohort; n = 25), or immune checkpoint inhibitor (ICI cohort; n = 5) treatment between August 2014 and July 2020 at the Department of Urology at Semmelweis University (Table 1). Three patients were included in both the RNU and CTX cohort and one patient in all three (RNU, CTX, and ICI) treatment groups, while two patients were included both in CTX and ICI cohorts. For the RNU cohort, in addition to the 34 preoperatively collected serum samples, 16 postoperative (first or second day) samples were available. For the CTX cohort, in addition to the 25 baseline samples, 18 on-treatment serum samples (collected at the start of the second chemotherapy cycle) were available. For all ICI-treated patients, baseline and on-treatment samples (taken after 3 months of treatment) were available for analysis. In addition, preoperative serum samples from three patients with pT0 finding at RNU were also included (Table 1). The primary endpoint of this study was overall survival (OS) and the secondary endpoint was progression-free survival (PFS). Time to disease progression and death or survival were considered as the time from the initiation of therapy (RNU, CTX, or ICI) to the relevant endpoint (progression or death) or censoring. The date of last follow-up update was June 2021.

This study was conducted in accordance with the Declaration of Helsinki, and the institutional ethics committee approved the study protocol (TUKEB 256/2014). All patients provided written informed consent to participate in this study.

Blood samples were collected in 9 mL Vacuette^®^ tubes. Samples were left at room temperature for 30–90 min and were separated via centrifugation for 10 min at room temperature with an Eppendorf 5702R centrifuge at 1500× *g*. Serum samples were aliquoted into anonymized tubes and kept at −80 °C until analysis.

### 2.2. Serum MMP-7 ELISA Analysis

MMP-7 serum concentrations were determined by the sandwich ELISA (Enzyme-linked immunosorbent assay) method using the Human Total MMP-7 Quantikine ELISA kit (R&D Systems, Wiesbaden, Germany, Catalog Number: DMP700), according to the product instructions. Colorimetric detection was performed by a Thermo Scientific™ Multiscan FC Microplate Photometer. The results were analysed with the help of Skanlt 5.0 Software.

### 2.3. Statistical Analysis

The nonparametric two-sided Wilcoxon rank sum test (Mann–Whitney test) was applied for paired group comparisons. Univariate overall and progression-free analyses were done using both Kaplan–Meier log-rank test and univariate Cox analysis. To determine the optimal cut-off value with the highest sensitivity and specificity for the prediction of patients’ death, we used the nonparametric receiver operating characteristic (ROC) curves. In all tests, a *p*-value of at least 0.05 was considered to be significant. All statistical analyses were done with the IBM SPSS Statistics for Windows, version 27.0 (IBM Corp., Armonk, NY, USA).

## 3. Results

### 3.1. Clinical Background

Patient characteristics for the RNU, CTX, and ICI cohorts and baseline MMP-7 concentrations are given in Table 1.

The median ages at treatment in the RNU, CTX, and ICI groups were 69, 71, and 65 years, while the median follow-up times were 24, 17, and 28 months, respectively.

### 3.2. Correlation of Serum MMP-7 Concentrations with Clinicopathological Parameters

**Figure 1:**
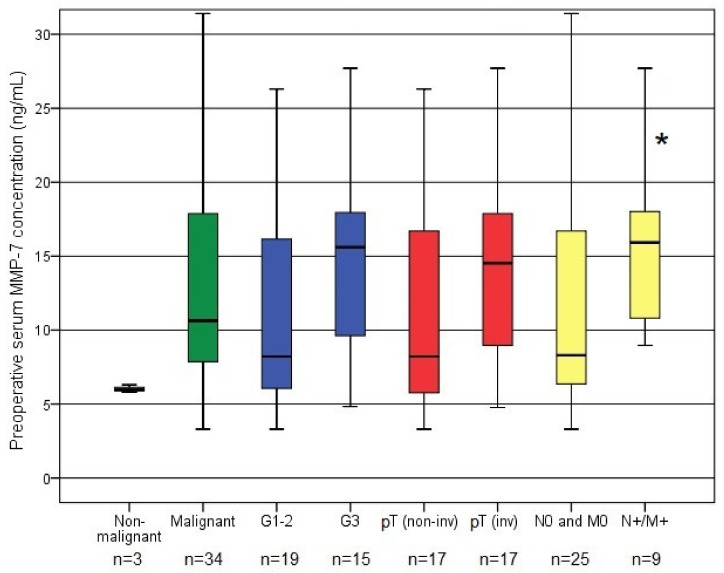
Association of preoperative serum MMP-7 concentration and clinicopathological parameters in the RNU cohort (* significant difference).

For the RNU cohort, the preoperative serum MMP-7 levels tended to be lower in pT0 cases compared to those present with confirmed tumour cells in the removed tissue (5.96 ng/mL vs. 10.63 ng/mL), however this correlation did not reach statistical significance (*p* = 0.071). Preoperative serum MMP-7 levels showed no association with age, gender, ECOG performance status, or margin positivity. Higher serum MMP-7 levels were found in muscle-invasive and high-grade tumours, but these correlations were not significant (*p* = 0.140 and *p* = 0.077). In addition, the presence of lymph node or distant metastases at surgery was significantly associated with higher preoperative serum MMP-7 levels (*p* = 0.045) (Table 1 and Figure 1).

For the CTX cohort, age, sex, and clinicopathological parameters at RNU were not correlated with pre-treatment MMP-7 levels. In contrast, the presence of distant metastases at chemotherapy baseline was associated with higher MMP-7 concentrations (7.8 ng/mL vs. 18.6 ng/mL; *p* = 0.040).

For the ICI cohort, the low case numbers did not allow for a valid statistical analysis, therefore the cohort characteristics are provided on an individual patient level in Table 2.

### 3.3. Correlation of Pre-Treatment Serum MMP-7 Levels with Patients’ Prognosis

In the RNU cohort, Cox univariate analysis revealed a shorter OS in high-stage (≥pT2) (HR: 7.115; 95% CI 1.504–33.659; *p* = 0.013), high-grade (G3) (HR: 5.060; 95% CI 1.325–19.323; *p* = 0.018), and lymph node or distant metastatic (HR: 4.891; 95% CI 1.379–17.345; *p* = 0.014) UTUC patients. Similarly, poor PFS was associated with the same three factors (T-stage—HR: 23.899; 95% CI 3.087–185.035; *p* = 0.002; grade—HR: 7.670; 95% CI 2.114–27.833; *p* = 0.002; lymphatic or distant metastases—HR: 8.250; 95% CI 2.742–24.820; *p* < 0.001) (Table 3).

**Figure 2:**
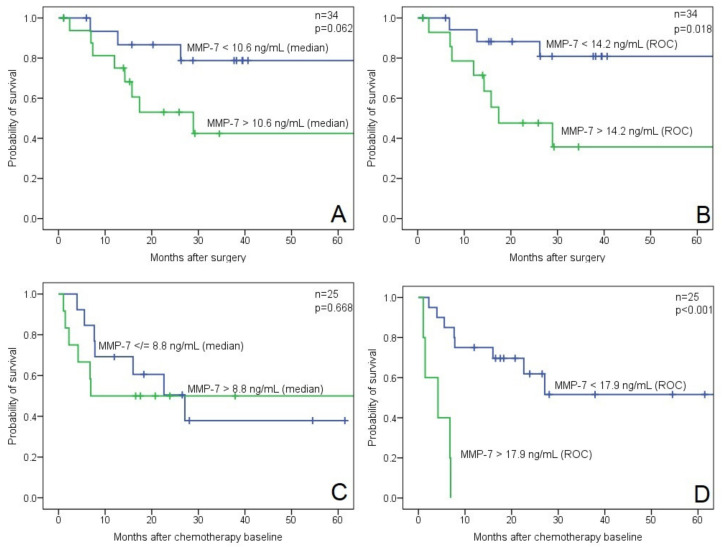
Kaplan–Meier OS analyses with log-rank tests (**A**) for the RNU cohort with a median value, (**B**) for the RNU cohort with an ROC cut-off, (**C**) for CTX cohort with a median value, and (**D**) for the CTX cohort with an ROC cut-off (blue line—low MMP-7 cc., green line—high MMP-7 cc.; cut-off values are shown on each line).

Patients were divided into two groups based on their pre-treatment MMP-7 concentrations, using the median value as a cut-off (10.625 ng/mL) (Figure 2A). In addition, we used the receiver operating characteristic (ROC) method for the prediction of OS to find the cut-off value with the highest sensitivity (72.7%) and specificity (69.6%), which resulted in 14.185 ng/mL as an optimal cut-off (Figure 2B). Higher preoperative serum MMP-7 levels were associated with poor OS; however, this correlation was significant only at the ROC-defined cut-off (*p* = 0.029) and slightly missed the significance level when using the median as a cut-off (*p* = 0.078) (Table 3).

In the CTX cohort, the presence of lymph node or distant metastases at chemotherapy baseline was significantly associated with poor OS and PFS (*p* = 0.017 and *p* = 0.002, respectively) (Table 3). Higher baseline MMP-7 levels were only associated with shorter OS (*p* < 0.001) when the ROC defined cut-off value of 17.88 ng/mL was applied (Figure 2D). This high cut-off, however, has to be handled with caution, as only five patients were assigned in the high MMP-7 group (Table 3). The relative low number of observed events (deaths) in each subgroup (RNU: 11, CTX: 13, and ICI: 1) did not allow us to perform multivariate survival analyses.

### 3.4. Postoperative and On-Treatment Serum MMP-7 Levels

**Figure 3:**
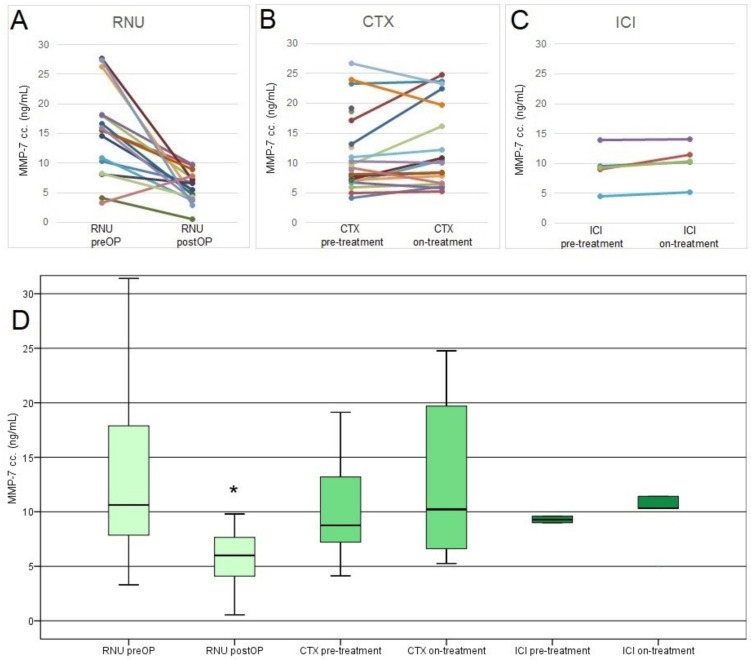
(**A**) Changes of MMP-7 concentrations in the RNU cohort (preop. and postop. values). (**B**) Changes of MMP-7 concentrations in the CTX cohort (at chemotherapy baseline and on the first day of cycle 2). (**C**) Changes of MMP-7 concentrations in the ICI cohort (pre-treatment and on-treatment values). (**D**) Changes of MMP-7 concentrations in the RNU, CTX, and ICI cohorts (* significant difference); RNU—radical nephroureterectomy; CTX—chemotherapy; ICI—immune checkpoint inhibitor therapy.

In the RNU cohort, the median preoperative serum MMP-7 level of 10.6 ng/mL significantly decreased to 6 ng/mL (*p* < 0.001), suggesting that MMP-7 levels are directly associated with the presence of UTUC (Figure 3A,D). However, the extent of the postoperative MMP-7 decrease was not associated with patients‘ prognosis. 

In the CTX cohort, we found no significant changes of serum MMP-7 levels between baseline and the start of second therapy cycle, and individual changes of serum MMP-7 during the first chemotherapy cycle were not associated with patients’ prognosis (Figure 3B,D).

In ICI treated patients, the on-treatment serum MMP-7 levels after 3 months of ICI treatment were slightly higher in all five cases, however here, low case numbers did not allow us to draw statistically firm conclusions (Table 2).

## 4. Discussion

In the present study, we analysed the serum MMP-7 concentrations in three treatment cohorts (RNU, CTX and ICI) of UTUC patients and found significantly elevated pre-treatment MMP-7 levels in patients with lymph node or distant metastases. Importantly, high pre-treatment MMP-7 levels were significantly associated with poor patients’ survival in the RNU and CTX cohorts.

UTUC is a clinically heterogenous disease with difficult prognostication due to the limited accuracy of biopsy for the evaluation of the tumour stage [13]. Therefore, in recent years, a significant effort has been invested towards the improvement of risk-stratification by preoperatively available parameters such as imaging and biomarkers [14,15]. However, most of the suggested models contain histology-based parameters (T-stage, grade, lymphovascular invasion, and lymph node status), which are affected by the inaccuracy of biopsy and imaging. Other often used parameters such as age, smoking-status, BMI, and ECOG performance status do not necessarily reflect the clinical behaviour of the disease [16,17]. In addition, tissue markers like Ki67, p53, EGFR, and E-cadherin have been investigated in smaller case series with promising results, however the relevance of these analyses is strongly limited by the availability of the biopsy specimen [15]. Against this background, serum biomarkers would be highly preferable in the prognostication of UTUC, as they are also easily available before surgical treatment and therefore may help to influence clinical decision-making regarding the extent of surgical treatment or the application of neoadjuvant systemic therapy. However, to the best of our knowledge, besides routinely determined laboratory parameters, no blood-based prognostic biomarkers have been described in UTUC [15].

In BC, we previously identified two-fold significantly higher tissue MMP-7 expression in muscle-invasive compared to non-muscle-invasive bladder cancer samples, while the highest 30x elevated tissue MMP-7 expression levels were found in the primary tumours tissues of patients present with lymph node metastases. Accordingly, serum and urine MMP-7 levels showed significantly higher levels in high-stage and metastatic cases. Furthermore, the presence of lymph node or distant metastases and high MMP-7 concentrations were independently associated with a shorter postoperative survival [7]. These observations were later confirmed in three independent BC cohorts with an overall number of more than 300 BC patients [9,10,11]. In a further study, 422 protein markers were determined in serum samples of BC patients, and MMP-7 was one of the five markers found to be prognostic [7]. In the present study, we found a significantly elevated MMP-7 concentration of 15.9 ng/mL in metastatic UTUC, which was similar to that of 13.9 n/mL that was previously found in metastatic BC [7]. Similar to the findings in BC, in the present study, we found preoperative serum MMP-7 levels to be associated with shorter survival in UTUC patients, suggesting that serum MMP-7 may help to improve the preoperative prognostication of this disease. Of note, in three cases of RNU, histological evaluation resulted in a pT0 finding, and the preoperative MMP-7 concentrations in all of these cases were proven to be low (5.9 ng/mL), suggesting that serum MMP-7, when combined with other factors, e.g., imaging and laboratory parameters, may help to select patients for an organ sparing treatment strategy. A further similarity to the findings in BC was the significant decrease of serum MMP-7 (from 10.6 to 6.0 ng/mL) directly after RNU. This decrease suggests that circulating MMP-7 levels directly originate from the tumour tissue. On the other hand, the extent of MMP-7 decrease was not associated with patients’ survival. However, we were not able to analyse MMP-7 tissue expression in the present UTUC cohort, and based on the histological similarity between UTUC and BC, we assume that the tissue expression pattern of MMP-7 in UTUC may be similar to that found in BC.

Previously published studies revealed the role of MMP-7 in chemotherapy (including cisplatin) resistance in various cancers [18,19,20,21]. In line with these findings, in BC, higher MMP-7 concentrations before platinum therapy were significantly associated with poor overall survival [22]. Similarly, in UTUC patients, we found a correlation between pre-treatment MMP-7 concentrations and shorter OS. However, in contrast to the findings in the RNU cohort, serum MMP-7 did not change significantly after the first cycle of platinum chemotherapy, however later samples from later therapy cycles were not tested yet. In the five ICI-treated patients, we observed a slight decrease of MMP-7 levels in on-treatment samples after 3 months of therapy compared to the corresponding baseline levels, however this change did not reach the significance level. In summary, MMP-7 is a serum marker that seems to be directly associated with the presence of highly aggressive tumours, and represents a promising preoperative prognostic marker for UTUC. Our results, however, need to be confirmed in larger patient cohorts to overcome the limitations inherent from the retrospective nature and the relatively small sample size of this study. This limitation, however, should be judged in relation to the low incidence of this rare disease. A further limitation is that we could not compare our data with imaging and next generation imaging methods.

A deeper insight into the role of MMP-7 in molecular and cellular mechanisms is needed for a better understanding of the role of MMP-7 in the formation and progression of various cancers and for its critical evaluation as a potential therapeutic target. MMP-7 is a protease with a broad substrate specificity, which is thereby involved in several molecular processes. As an example, degradation of Fas and Fas ligand by MMP-7 was shown to result in the inhibition of apoptosis [20,23]. Similarly, by degrading the extracellular domain of the epithelial gap junction molecule, E-cadherin, MMP-7 was shown to support epithelial-to-mesenchymal transition, which is a frequently observed phenomenon during the progression of epithelial cancers [24]. On the other hand, the products of the proteolytic degradation by MMP-7 may generate bioactive molecules with potential effects on tumour progression. For example, MMP-7, by degrading the extracellular matrix protein collagen XVIII, is able to release endostatin, a potent antiangiogenic factor, thereby influencing angiogenesis. [25]. A further example for the proteolytic activation effect of MMP-7 lies in its role as an activator of other MMP family members and activating a proteolytic cascade. MMP-7 is able to cleave the extracellular domain of some prominent proteins with known involvement in carcinogenesis, such as syndecan-1 and PD-L1 (Programmed death-ligand 1) [26,27,28]. Therefore, MMP-7 may influence epithelial cell adhesion and antitumor immunity [25,29]. Histological and molecular analyses suggest a focal distribution of MMP-7 with an accentuated expression at the invasive tumour front, suggesting its involvement in tumour infiltration [7]. Accordingly, Bolenz et al. showed that targeted downregulation of MMP-7 leads to a strong decrease in the invasive capability of BC cells [30]. Furthermore, comparing the gene expression patterns between the central tumour region and tumour bone interface in prostate cancer bone metastases revealed MMP-7 as one of the most strongly upregulated genes at the tumour–bone interface [31,32]. Accordingly, in an MMP-7 deficient rodent bone metastasis model, the absence of MMP-7 expression was associated with strongly reduced osteolytic activation and metastasis formation [31]. Later, a specific downregulation of MMP-7 in UTUC cells resulted in a decreased expression of epithelial-to-mesenchymal markers, as well as reduced motility and invasive potential of cells, highlighting the functional role of MMP-7 in UTUC [33]. Taken together, these findings suggest the functional involvement of MMP-7 in the invasion and metastatic progression of urological tumours, and make MMP-7 a potential target for therapy also in UTUC. Considering the failure of earlier prospective clinical trials with broad-spectrum MMP-inhibitors (batimastat and marimastat), an MMP subtype-specific therapeutic approach may be more feasible [34,35]. Currently, a selective MMP-7 inhibitor has been generated and holds promise for future MMP-7 targeted therapy [36].

## Figures and Tables

**Table 1:** Patients’ characteristics. * Non-malignant—in three cases of RNU, histological examination resulted in a pT0 finding; RNU—radical nephroureterectomy; CTX—chemotherapy; ICI—immune checkpoint inhibitor therapy; ECOG PS—Eastern Cooperative Oncology Group performance status; R+—positive surgical margin; N+—lymph node metastasis; M+—distant metastasis; bold font represents significant value.

	RNU			CTX			ICI	
General data	n	median (range)	*p*	n	median (range)	*p*	n	median (range)
Follow up in months, median (range)	34	24.23 (1.08–81.93)	-	25	16.56 (1.05–67.70)	-	5	27.97 (6.92–30.2)
Age at baseline, median (range)	34	68.50 (45–90)	-	25	71 (46–84)	-	5	65 (64–75)
Number of patients died	11	-	-	13	-	-	1	-
Parameters/MMP-7 concentrations	n	MMP-7 cc.	*p*	n	MMP-7 cc.	*p*	n	MMP-7 cc.
All patients, median (range)	34	10.63 (3.30–31.40)	0.071	25	8.76 (4.13–26.74)	-	5	9.28 (4.46–13.90)
Non-malignant *	3	5.96 (5.80–6.30)						
Age ≤ 65	10	7.37 (3.3–27.36)	0.086	5	7.3 (4.13–9.17)	0.129	2	9.14 (9.00–9.28)
Age > 65	24	14.19 (4.05–31.40)		20	9.99 (4.92–26.74)		3	9.60 (4.46–13.90)
Sex male	21	10.3 (3.3–27.36)	0.381	21	7.91 (4.13–26.74)	0.081	4	9.14 (4.46–13.90)
female	13	13.84 (4.84–31.40)		4	12.93 (10.28–23.28)		1	9.60
ECOG PS 0	19	13.84 (3.30–27.70)	-	11	8.08 (5.86–17.18)	-	5	9.28 (4.46–13.90)
1	10	9.32 (4.77–31.40)	-	10	10.91 (4.13–26.74)	-	0	
2	4	9.89 (8.08–17.00)	-	4	13.18 (6.68–23.28)	-	0	
3	1	15.92		0			0	
ECOG PS 0–1	29	10.44 (3.30–31.40)	0.888	21	8.76 (4.13–26.74)	0.695	5	9.28 (4.46–13.90)
ECOG PS 2–3	5	10.81 (8.08–17.00)		4	13.18 (6.68–23.28)		0	
Nephrouretherectomy data								
pTa	7	9.80 (3.30–26.30)	-	0	-	-	0	-
CIS	1	4.84	-	0	-	-	1	13.90
pT1	9	8.21 (4.05–23.73)	-	1	19.13	-	0	-
pT2	2	22.97 (14.53–31.40)	-	5	8.76 (6.73–24.02)	-	1	9.00
pT3	14	10.63 (4.77–27.70)	-	15	7.91 (4.13–18.59)	-	3	9.28 (4.46–9.60)
pT4	1	17.88	-	2	7.31 (4.92–9.70)	-	0	-
n.a.	0			2		-	0	
pTa-pT1-CIS (non-invasive)	17	8.21 (3.30–26.30)	0.140	1	19.13	-	1	13.90
pT2-pT4 (invasive)	17	14.53 (4.77–31.40)		22	7.99 (4.13–24.02)	-	4	9.14 (4.46–9.60)
G1	7	8.06 (4.05–13.84)	-	0		-	0	
G2	12	13.03 (3.30–26.30)	-	5	13.2 (6.73–24.02)	-	2	9.14 (9.00–9.28)
G3	15	15.60 (4.84–31.40)	-	16	7.53 (4.13–18.59)	-	2	9.18 (4.46–13.90)
n.a.	0			4			1	
G1-G2	19	8.21 (3.30–26.30)	0.077	5	13.2 (6.73–24.02)	0.075	2	9.14 (9.00–9.28)
G3	15	15.60 (4.84–31.40)		16	7.53 (4.13–18.59)		2	9.18 (4.46–13.90)
R0	26	10.37 (3.30–31.40)	0.827	14	7.99 (4.13–24.02)	0.224	3	9.00 (4.46–9.60)
R+	8	12.67 (4.77–23.73)		9	8.76 (4.92–18.59)		1	9.28
n.a.	0			2			1	
Metastatic status at RNU								
N0/M0	25	8.30 (3.30–31.40)	**0.045**	14	7.60 (5.86–24.02)	0.781	2	11.45 (9.00–13.90)
N+ or M+	9	15.92 (8.96–27.70)		9	9.26 (4.13–19.13)		3	9.28 (4.46–9.60)
n.a.	0			2			0	
Metastatic status at CTX baseline								
M0	-			17	7.77 (4.13–17.18)	**0.040**	-	
M+	-			7	18.59 (6.68–26.74)		-	

**Table 2:** Patient’s characteristics for the ICI cohort. ICI—immune checkpoint inhibitor therapy; N —lymph node metastasis; M—distant metastasis; Gem/Carb—gemcitabine + carboplatin; Gem/Cis—gemcitabine + cisplatin; n.a.—not available.

	Patient 1	Patient 2	Patient 3	Patient 4	Patient 5
Age	76	64	64	75	65
Sex	female	male	male	male	male
Clinicopath. parameters at RNU					
Stage (pT)	3	2	3	in situ	3
Grade (G)	-	2	2	3	3
N	yes	no	yes	no	yes
M	no	no	no	no	no
Chemotherapy pre-treatment	Gem/Carb	Gem/Cis	Gem/Carb	n.a.	Gem/Carb
Clinicopath. parameters at ICI baseline					
N	yes	yes	yes	no	yes
M	yes	yes	no	no	yes
MMP-7 cc. at baseline (ng/mL)	9.60	9.00	9.28	13.90	4.46
MMP-7 cc. at 3 months (ng/mL)	10.30	11.42	10.33	14.13	5.12

**Table 3:** Correlation of clinicopathological parameters and preoperative MMP-7 concentrations with patients’ prognosis; * median cut-off for RNU is 10.6 ng/mL, median cut-off for CTX is 8.8 ng/mL; ** ROC cut-off for RNU is 14.2 ng/mL, ROC cut-off for CTX is 17.9 ng/mL; RNU—radical nephroureterectomy, CTX- chemotherapy; OS—overall survival; PFS—progression-free survival; PS—performance status; Gem/Cis—gemcitabine + cisplatin; Gem/Carb—gemcitabine + carboplatin; bold font represents significant value.

		RNU						CTX							
				OS			PFS			OS			PFS		
General data		n	HR	95% CI	*p*	HR	95% CI	*p*	n	HR	95% CI	*p*	HR	95% CI	*p*
Age	≤65	10	ref.			ref.			5	ref.			ref.		
	>65	24	2.142	0.459–9.994	0.332	1.775	0.494–6.378	0.379	20	1.675	0.370–7.584	0.503	0.552	0.171–1.783	0.321
Sex	male	21	ref.			ref.			21	ref.			ref.		
	female	13	0.281	0.060–1.301	0.104	0.330	0.091–1.191	0.090	4	0.374	0.049–2.884	0.345	0.337	0.044–2.579	0.295
ECOG PS before therapy	0–1	29	ref.			ref.			21	ref.			ref.		
	2–3	5	3.451	0.707–16.832	0.126	2.926	0.807–10.608	0.102	4	2 124	0.571–7.899	0.261	0.954	0.212–4.291	0.951
Nephrouretherectomy data															
Stage	pTa-pT1-CIS	17	ref.			ref.			1	ref.			ref.		
	pT2-pT4	17	7.115	1.504–33.659	**0.013**	23.899	3.087–185.035	**0.002**	22	1.000	0.0–14926.7	1.000	21.482	0.000–7.5 × 10^19^	0.888
Metastases	N0/M0	25	ref.			ref.			14	ref.			ref.		
	N+ or M+	9	4.891	1.379–17.345	**0.014**	8.250	2.742–24.820	**<0.001**	9	3.065	0.923–10.181	0.068	3.651	1.237–10.773	**0.019**
Grade	1–2	19	ref.			ref.			5	ref.			ref.		
	3	15	5.060	1.325–19.323	**0.018**	7.670	2.114–27.833	**0.002**	16	1.379	0.297–6.412	0.682	0.945	0.258–3.453	0.931
Chemotherapy baseline data															
Metastases	N0/M0	-	-			-			10	ref.			ref.		
	N+ or M+	-	-			-			14	6.722	1.410–32.049	**0.017**	8.985	2.309–34.961	**0.002**
Chemotherapy regimen:	Gem/Cis	-	-			-			14	ref.			ref.		
	Gem/Carb	-	-			-			11	0.907	0.296–2.777	0.864	1.893	0.658–5.448	0.237
Pretreatment serum MMP-7 level															
serum MMP-7	median cut-off *	17	ref.			ref.			13	ref.			ref.		
serum MMP-7	median cut-off *	17	3.324	0.874–12.644	0.078	1.694	0.585–4.900	0.331	12	1.270	0.425–3.797	0.669	0.799	0.267–2.394	0.689
serum MMP-7	ROC cut-off **	19	ref.			ref.			20	ref.			ref.		
serum MMP-7	ROC cut-off **	15	4.413	1.159–16.798	**0.029**	1.676	0.585–4.799	0.336	5	12.063	2.800–51.968	**0.001**	2.166	0.452–10.380	0.334

## Data Availability

The data that support the findings of this study are available from the corresponding author upon reasonable request.
